# A Time- and Space-Integrated Expansion Planning Method for AC/DC Hybrid Distribution Networks

**DOI:** 10.3390/s25072276

**Published:** 2025-04-03

**Authors:** Yao Guo, Shaorong Wang, Dezhi Chen

**Affiliations:** School of Electrical and Electronic Engineering, Huazhong University of Science and Technology, Wuhan 430074, China; d202080604@hust.edu.cn (Y.G.); dzhchen@hust.edu.cn (D.C.)

**Keywords:** AC/DC hybrid distribution network, expansion planning, graph attention network, deep reinforcement learning

## Abstract

The rapid growth of renewable energy and increasing electricity demand pose challenges to the reliability and flexibility of traditional distribution networks. To address these issues, the construction of AC/DC hybrid distribution networks (AC/DC-HDNs) based on existing AC grids has become a promising solution. However, planning the expansion of such networks faces challenges like complex device and line topologies, dynamic fluctuations in distributed generation (DG) and load, and high power electronics costs. This paper proposes a time- and space-integrated expansion planning method for AC/DC-HDNs. The approach builds a distribution grid model based on graph theory, integrating the spatial layouts of AC distribution lines, DGs, main grids, and loads, while capturing dynamic load and renewable energy generation characteristics through time-series analysis. A modified graph attention network (MGAT)-based deep reinforcement learning (DRL) algorithm is used for optimization, balancing economic and reliability objectives. The simulation results show that the modified algorithm outperforms traditional algorithm in terms of both training efficiency and stability, with a faster convergence and lower fluctuation in cumulative rewards. Additionally, the proposed algorithm consistently achieves higher cumulative rewards, demonstrating its effectiveness in optimizing the expansion planning of AC/DC-HDNs.

## 1. Introduction

With the rapid development of renewable energy and the continuous growth in electricity demand worldwide, traditional AC distribution networks are facing increasingly severe challenges in terms of the reliability and flexibility of the power supply [1]. The integration of renewable energy sources, such as wind and solar power, has not only diversified the energy mix but also introduced uncertainty and variability. This has further intensified the pressure on traditional distribution networks in terms of load management and power quality control. Consequently, a transformation of traditional distribution networks is urgently needed to adapt to the increasingly complex energy landscape and the ever-changing electricity demands [2].

Compared to traditional AC distribution networks, AC/DC hybrid distribution networks (AC/DC-HDNs) effectively integrate multiple energy sources by incorporating converters and DC lines, enhancing their compatibility with DC loads and renewable energy. Furthermore, AC/DC-HDNs enable multi-energy interconnection and regional energy optimization [3]. However, due to the significant increase in complexity related to network topology, power flow control, and operational optimization [4], a key challenge for widespread application lies in how to optimally plan the integration of new energy sources and the construction of DC lines based on the existing distribution network.

With the large-scale integration of distributed energy sources, such as wind and solar power, the dynamic characteristics of distribution networks have become increasingly pronounced. Traditional static optimization methods often overlook the time-varying nature of load demand and generation [5,6]. Recent studies have explored approaches to address this issue. Reference [7] integrates demand response (DR) and distributed generation (DG) into distribution network planning, improving the flexibility and reducing costs, while reference [8] focuses on optimal battery storage system (BSS) planning, considering degradation over time to enhance the long-term scheduling efficiency. These advancements highlight the increasing research emphasis on accounting for the dynamic variations in both electricity demand and generation in AC/DC-HDN planning [9].

In addition to challenges in the time dimension, AC/DC-HDN planning also faces issues in the spatial dimension. As renewable energy power stations continue to expand, their geographical distribution and integration with the existing distribution network directly impact the grid’s operational efficiency and stability [10]. Reference [11] adopted a GIS-based approach to optimize network planning, incorporating an iterative clustering method that accounts for spatial uncertainties in PV, wind turbines (WTs), and load distribution. Meanwhile, [12] has proposed a coordinated planning model for large-scale wind farms and energy storage, considering decision-dependent uncertainty (DDU) in wind power prediction, which affects spatial expansion decisions. These studies highlight the necessity of carefully selecting optimal integration points for renewable energy and strategically designing the transmission and conversion infrastructure [13,14]. Therefore, future AC/DC-HDN planning must consider dynamic changes in both the time and spatial dimensions, proposing more intelligent and flexible optimization methods to address the complex fluctuations in energy supply and demand [15].

In the existing research, traditional optimization methods such as Mixed Integer Programming (MIP) and the Genetic Algorithm (GA) are widely applied to distribution network structure optimization [12,13,14,15,16,17,18,19,20]. While these methods perform well in specific scenarios, they often struggle to accurately model the complex topology of AC/DC-HDNs, limiting their effectiveness in capturing key spatial and temporal characteristics. Moreover, as distribution networks expand, these methods face increasing computational challenges when incorporating dynamic variations in load demand and renewable energy generation over the planning horizon. To enhance the accuracy and adaptability of planning, recent studies have focused on integrating spatiotemporal features into AC/DC-HDN planning, allowing for more precise long-term decision-making under varying operational conditions.

To address the aforementioned challenges, this paper proposes an expansion planning method for AC/DC-HDNs by integrating temporal and spatial information. The proposed method first constructs a graph-based model to represent the spatial topology of the network and incorporates time-series analysis to capture the dynamic characteristics of the load demand and renewable energy generation, aiming to optimize the network expansion plan. The optimization objectives are to enhance the accommodation of renewable energy and reduce the power loss of the distribution network. To achieve these goals, a comprehensive optimization algorithm combining a modified graph attention network (MGAT) and deep reinforcement learning (DRL) is introduced, enabling intelligent decision-making for resource allocation and network topology expansion in complex system environments. The main contributions of this paper are as follows:

(1) This paper proposes a graph-based expansion planning method for AC/DC-HDNs that integrates temporal and spatial information. It incorporates the network’s spatial layout and uses time-series analysis to capture the load and renewable generation dynamics, enhancing the flexibility and accuracy of planning to manage renewable energy uncertainties.
(2) This paper proposes three types of expansion lines for AC/DC-HDNs: bidirectional AC lines, bidirectional DC lines, and unidirectional DC lines. Bidirectional DC lines support frequent power exchanges, while unidirectional DC lines suit a stable, single-direction flow, reducing power loss and improving the power utilization efficiency. The simulation results show that the optimized networks improved renewable energy consumption by 3.64%, 3.31%, and 2.77% for IEEE 33-bus, IEEE 69-bus, and PG&E 69-bus systems, respectively.
(3) This paper integrates MGAT and DRL into a unified hybrid algorithm for dynamic optimization in AC/DC-HDNs. By incorporating edge features, the hybrid algorithm enhances the topology understanding and decision accuracy, enabling efficient optimization in complex spatiotemporal environments. The simulation results demonstrate that the hybrid algorithm achieves higher reward values and better convergence performance.

The paper is structured as follows: Section 2 presents the graph-based model and defines the AC/DC-HDN expansion planning problem. Section 3 introduces the optimization algorithm combining MGAT and DRL. Section 4 shows the simulation results, and Section 5 concludes with the main contributions and future research directions.

## 2. Graph-Based Model of AC/DC-HDN and Definition of Expansion Planning Problem

The distribution network is a core component of modern power systems, featuring a complex topology and dynamic changes in energy supply and electricity demand. In the expansion planning of AC/DC-HDNs, accurate modeling is essential for ensuring the efficient and reliable operation of the grid. Graph theory is widely used in power system modeling and optimization, because it can effectively represent the network’s topology and power flow. This section presents the graph-based modeling method for the AC/DC-HDN and defines the optimization objectives in the network expansion planning problem.

### 2.1. Construction of the Graph Model for AC/DC-HDN

In graph theory, a graph consists of vertices (nodes) and edges (lines) connecting them. This model is used to represent relationships between entities, with vertices denoting the entities and edges indicating their connections. Given its clear topology, a distribution network is naturally suited to a graph-based model. Typically, the main components of the network are represented as vertices, with edges depicting their connections or dependencies. In this section, however, the graph model for the AC/DC-HDN is redefined.

**Figure 1:**
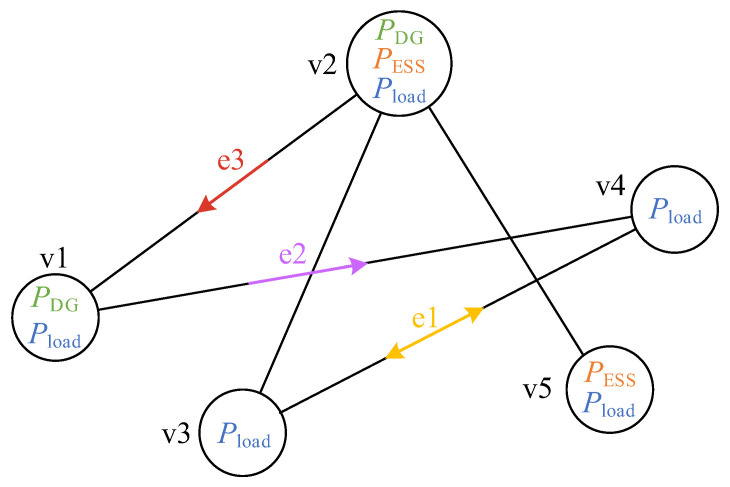
AC/DC-HDN graph model.

To effectively model the AC/DC-HDN, graph theory is used to abstract it into a graph $G=\left(V,E\right)$ with *I* nodes, as shown in Figure 1 ($v_{1},\ldots ,v_{5}\in V$ and $e_{1},e_{2},e_{3}\in E$).

This graph consists of a set of vertices, *V,* and a set of directed edges, *E*, defined in the following paragraphs.

**Figure 2:**
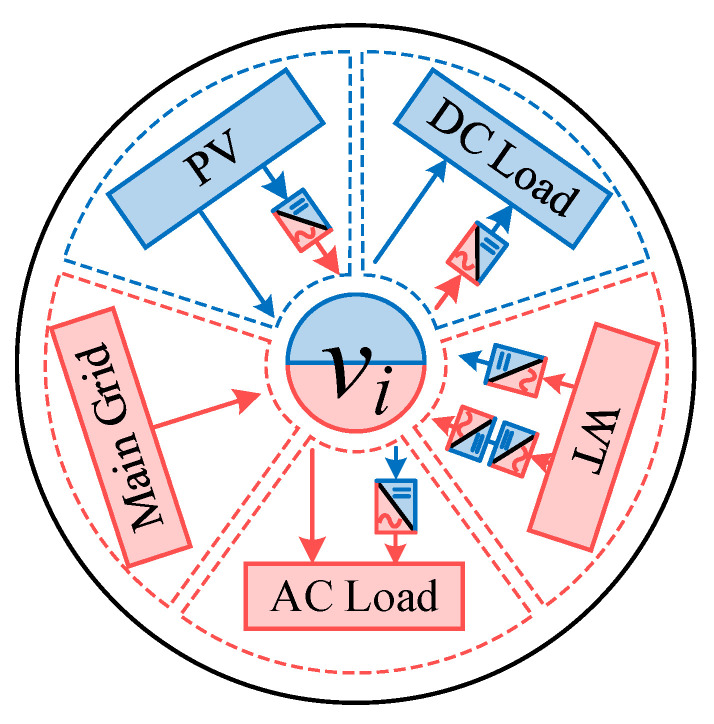
Vertex in the AC/DC-HDN graph model.

**The vertex set *V*:** Each vertex in the graph represents a power node, as shown in Figure 2. The injected power at each node includes three types: renewable energy generation power, power supplied from the main grid, and load demand power. These nodes are generally considered as switching stations in the distribution network.

**Figure 3:**
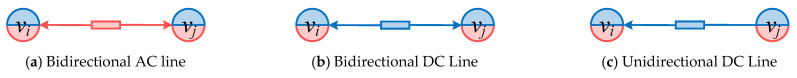
Edges in the AC/DC-HDN graph model.

**The edge set *E*:** Each edge in the graph represents a power flow path from one vertex to another. The edges can be classified into three types as shown in Figure 3, each representing a different type of line and power flow direction.

1. Bidirectional AC Line: This line represents a connection between two vertices through a switch device, allowing for a bidirectional flow of the alternating current. In the expansion planning of the AC/DC-HDN, this type of edge is typically defined as an existing AC line within the distribution network.
2. Bidirectional DC Line: This line represents a connection between two vertices through a bidirectional converter, which is embedded within the vertex, allowing for a bidirectional flow of the direct current. This type of edge facilitates the scheduling and control of the DC power flow, making it suitable for interconnecting DC loads. It is a key component to plan and construct in the expansion of the network.
3. Unidirectional DC Line: This line represents a connection between two nodes through a unidirectional converter, which is embedded within the vertex, allowing the power to flow in only one direction. This type of edge is typically used in scenarios where the power flows in a single direction, such as transmitting electricity from a distributed energy source to a load. It is a key component that requires planning and construction.

This line-type-based topology design offers several advantages for expanding AC/DC-HDNs. First, by optimizing the ratio of bidirectional to unidirectional DC lines, the network topology can be improved. Bidirectional DC lines are ideal for areas with frequent power exchanges, enhancing the system flexibility, while unidirectional DC lines are better for stable power flows. This approach provides greater flexibility in planning complex distribution networks. Second, bidirectional DC lines allow for flexible power flow control, boosting system reliability and fault recovery. Unidirectional DC lines, on the other hand, optimize the power flow and reduce network losses by preventing reverse power flow. This integrated design improves the overall operational efficiency of the network.

### 2.2. Description of the Expansion Planning Optimization Problem

Planning the expansion of an AC/DC-HDN is a complex, multidimensional decision-making process. The primary challenge lies in how to expand the existing AC distribution grid to incorporate AC/DC hybrid components. This planning process involves two key decisions: the optimal locations for renewable energy plants and the selection of DC transmission line types and placements. Moreover, the optimization goal is not only to ensure the security and stability of the power system, but also to maximize renewable energy absorption and optimize the resource allocation.

The expansion planning method for AC/DC-HDNs proposed in this paper aims to optimize the system’s operational efficiency by rationally planning the integration points of distributed energy sources and the types and layout of lines. This is achieved primarily by maximizing renewable energy consumption and minimizing network power losses, which can be expressed as follows:

(1) $$C_{2}=\min\left(\sum P^{\mathrm{MG}}+\sum P^{\mathrm{loss}}\right)$$

where $\sum P^{\mathrm{MG}}$ represents the total power supplied by the main grid, and $\sum P^{\mathrm{loss}}$ represents the total power loss in the AC/DC-HDN lines.

To ensure the feasibility and reliability of the expansion planning, the following constraints are considered in the optimization model:

Power Balance Constraint: The total power generation, including renewable energy sources and the upper-level grid supply, must match the total load demand plus system losses at all times:

(2) $$\sum P^{\mathrm{DG}}+\sum P^{\mathrm{MG}}=\sum P^{\mathrm{load}}+\sum P^{\mathrm{loss}}$$

Voltage Limit Constraint: The node voltages in both AC and DC networks must remain within allowable limits to ensure stable operation:

(3) $$V_{\min}\leq V_{i}\leq V_{\max},\;\forall i\in \mathrm{vertices}$$

Renewable Energy Penetration Constraint: The integration of distributed energy sources should maximize renewable energy utilization while ensuring grid stability:

(4) $$\sum P^{\mathrm{DG}}\geq \eta_{\min}\left(\sum P^{\mathrm{load}}+\sum P^{\mathrm{loss}}\right)$$

## 3. Optimization Algorithm Based on the Combination of MGAT and DRL

To efficiently solve the multi-objective optimization problem, this paper proposes a cooperative optimization framework combining an MGAT with the Proximal Policy Optimization (PPO) algorithm. The framework integrates spatiotemporal features to optimize the integrated placement of DGs and the configuration of DC lines. This section details the MGAT’s structure, its optimization mechanism, and the integration principle with the PPO algorithm for collaborative optimization.

### 3.1. Application of the MGAT

To process spatial information in the distribution network, a graph-based expansion planning model for the AC/DC-HDN is developed, providing a solid mathematical foundation for applying an MGAT in the optimization algorithm.

Graph Convolutional Networks (GCNs) are widely used for learning node features from graph-structured data through message passing between nodes. However, a GCN aggregates neighboring node information using a fixed adjacency matrix, which overlooks the varying importance of different neighbors and ignores edge features. In real-world scenarios, neighboring nodes influence the target node differently, making it necessary to assign dynamic weights to them. A GAT addresses this limitation by using a self-attention mechanism to dynamically assign different weights to each edge, capturing the varying significance of neighboring nodes more effectively. To further enhance the GAT’s performance in our proposed model, an edge feature matrix in the attention calculation is introduced. This allows the algorithm to accurately learn the characteristics of the three types of edges in the network.

The input features for the proposed MGAT are defined as follows:

- **Vertex Features:** In the MGAT model, each vertex feature is represented as a vector. According to Section 2.1, this feature vector is constructed from the actual input power data of each vertex and serves as the input to the network. Specifically, the feature $X_{i,t}$ of vertex $v_{i}\left(i\in I\right)$ at time *t* is a vector composed of renewable generation power, main grid power, and load demand, as shown in Equation (9).

(5) $$X_{i,t}=\left[\sum P_{i,t}^{\mathrm{DG}},\sum P_{i,t}^{\mathrm{MG}},-\sum P_{i,t}^{\mathrm{Load}}\right]$$

- **Edge Features:** For edge features in the graph, each edge is defined as a three-dimensional vector containing information on the existence, type, and length of the edge. The feature $Y_{\left(i,j\right),t}$ of edge $e_{\left(i,j\right)}\left(j\in I\right)$ at time *t* is represented as shown in Equation (10).

(6) $$Y_{\left(i,j\right),t}=\left[\alpha_{\left(i,j\right),t},\beta_{\left(i,j\right),t},l_{\left(i,j\right),t}\right]$$

where $a_{\left(i,j\right),t}\in \left\{0,1\right\}$. If $\alpha_{\left(i,j\right),t}=0$, the edge $e_{\left(i,j\right)}$ does not exist. If $\alpha_{\left(i,j\right),t}=1$, the edge $e_{\left(i,j\right)}$ exists. $\beta_{\left(i,j\right),t}\in \left\{-1,0,1\right\}$. If $\beta_{\left(i,j\right),t}=-1$, the edge is a bidirectional AC line. If $\beta_{\left(i,j\right),t}=0$, the edge is a bidirectional DC line. If $\beta_{\left(i,j\right),t}=1$, the edge is a unidirectional DC line. $l_{\left(i,j\right),t}$ represents the length of the edge $e_{\left(i,j\right)}$. When the edge does not exist ($\alpha_{\left(i,j\right),t}=0$), both the type and length information is represented as −2, i.e., $\beta_{\left(i,j\right),t}=l_{\left(i,j\right),t}=-2$.

**Figure 4:**
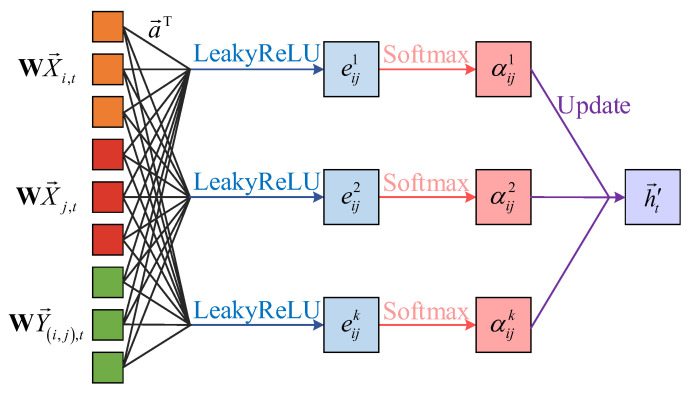
MGAT mechanism.

The core of the MGAT is the attention mechanism, which incorporates edge features to compute the relationships between nodes. Unlike traditional neural networks, where each node updates independently, the MGAT updates a node’s features by considering both its neighbors and the edge features. It calculates attention weights between nodes and their neighbors, using these weights to update both the node and edge features. This process enables the MGAT to better capture structural information from both nodes and edges, as shown in Figure 4.

For a vertex $v_{i}$ in the graph, the MGAT calculates an attention coefficient $e_{ij}$ based on the relationship between $v_{i}$ and its neighboring vertex $v_{j}$, representing the influence of $v_{j}$ on $v_{i}$. To incorporate edge features, the attention coefficient calculation is modified so that it is influenced not only by vertex features but also by edge features. Specifically, the attention coefficient $e_{ij}$ can be computed using the following function:

(7) $$e_{ij}=\mathrm{LeakyReLU}\left({\vec{a}}^{T}\left[W{\vec{X}}_{i,t}\left\|W{\vec{X}}_{j,t}\right.\left\|W{\vec{Y}}_{\left(i,j\right),t}\right.\right]\right)$$

where ${\vec{X}}_{i,t}$ and ${\vec{X}}_{j,t}$ are the feature vectors of vertex $v_{i}$ and $v_{j}$, respectively; ${\vec{Y}}_{\left(i,j\right),t}$ is the feature vector of edge $e_{\left(i,j\right)}$; W is a linear transformation matrix used to map vertex and edge features to a new space; ${\vec{a}}^{T}$ is the attention weight used to compute the correlation between nodes; ∥ denotes the vector concatenation operation; and LeakyReLU is the activation function.

After the feature weighting and concatenation operation, the attention coefficient $e_{\left(i,j\right)}$ for each vertex needs to be normalized to obtain the aggregation coefficient $\alpha_{ij}$. Then, the MGAT will perform a weighted aggregation of these coefficients, resulting in the updated features for $v_{i}$ and $e_{\left(i,j\right)}$. To improve the model’s stability and expressive power, the MGAT uses a multi-head attention mechanism. For each pair of vertices $v_{i}$ and $v_{j}$, the network calculates *K* independent attention coefficients $\alpha_{ij}^{k}$. These different coefficients produce distinct features, which are then concatenated to form a feature set representing the updated vertex or edge features:

(8) $${\vec{h}}_{i,t}^{\prime }=\overset{K}{\underset{k=1}{∥}}\sigma \left(\sum_{j\in N_{j}}\alpha_{ij}^{k}W{\vec{X}}_{j,t}∥W{\vec{Y}}_{\left(i,j\right),t}\right)$$

(9) $${\vec{h}}_{(i,j),t}^{\prime }=\overset{K}{\underset{k=1}{∥}}\sigma \left(\sum_{j\in N_{j}}\alpha_{ij}^{k}W{\vec{X}}_{i,t}∥W{\vec{X}}_{j,t}\right)$$

In the proposed AC/DC-HDN model, the vertex features include power data, while the edge features consist of the edge type, length, and connection status. These features may have varying impacts on the objective at different optimization stages. The multi-head attention mechanism dynamically assigns different weights to neighboring vertices based on these features, allowing for more precise information aggregation.

**Figure 5:**
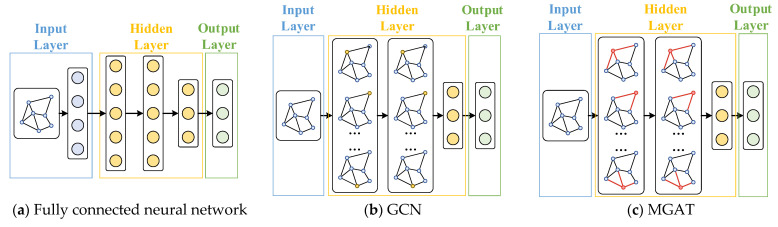
Comparison of neural network structures.

Figure 5 compares the structures of the MGAT, fully connected neural network, and GCN. The fully connected network flattens the data into a one-dimensional vector, losing the graph’s structural information. The GCN only considers vertex features and uses an adjacency matrix that reflects connectivity, missing some graph data. In contrast, the MGAT directly uses graph data as input, preserving all vertex and edge information, which significantly improves the model’s spatial awareness. This makes it more effective for tasks like planning the expansion of AC/DC-HDNs.

### 3.2. Application of the PPO

The MGAT effectively captures both vertex features and the topological relationships in the grid. Meanwhile, DRL leverages temporal information to learn optimal strategies through continuous interaction with the environment. Combining DRL with the MGAT is an ideal approach for optimizing the expansion planning of AC/DC-HDNs with spatial and temporal data.

In DRL, the optimization problem can be modeled as a Markov Decision Process (MDP), which includes the following elements:

1. **STATE**: The state represents the grid’s operating condition at a specific time, including vertex features (e.g., power information) and edge features (e.g., type, length, and existence). At each time step *t*, the state $s_{t}$ is defined as follows:
  (10) $$s_{t}=\left(P_{t}^{\mathrm{DG}},P_{t}^{\mathrm{MG}},-P_{t}^{\mathrm{Load}},\alpha_{t},\beta_{t},l_{t}\right)$$

The vectors $P_{t}^{\mathrm{DG}}$, $P_{t}^{\mathrm{MG}}$, and $-P_{t}^{\mathrm{Load}}$ represent the power of DGs, main grid supply, and load demand at each vertex. The vectors $\alpha_{t}$, $\beta_{t}$, and $l_{t}$ represent the connection status, type, and length of each edge in the AC/DC-HDN.

2. **ACTION:** The action space $a_{t}$ consists of the DG connection locations, and the existence attributes and types of all edges, represented as follows:

(11) $$a_{t}=\left(i_{t},\alpha_{t},\beta_{t}\right)$$

where $i_{t}$ is the vertex for DGs connection.

3. **REWARD:** The immediate reward $r_{t}$ reflects the feedback given to the agent at time step *t* after it takes the action $a_{t}$, causing the system to transition from state $s_{t}$ to $s_{t+1}$. As described in Section 2.2, the goal of the proposed AC/DC-HDN expansion planning method is to maximize the operational efficiency by optimizing the placement of renewable energy sources and the design of DC lines. The reward $r_{t}$ is expressed as follows:

(12) $$r_{t}=-\left(\sum P^{\mathrm{MG}}+\sum P^{\mathrm{loss}}\right)-k$$

where *k* is a penalty term that can be used to correct an improper action strategy. For the AC/DC-HDN, voltage violation is not permitted. Thus, *k* is set to be the following:

(13) $$k=\left\{\begin{array}{l} q\left(U_{i,t}-1.05U_{Ni}\right)\begin{array}{c} \;1.05U_{Ni}<U_{i,t} \end{array}\\ q\left(0.95U_{Ni}-U_{i,t}\right)\begin{array}{c} \;U_{i,t}\leq 0.95U_{Ni} \end{array} \end{array}\right.$$

where *q* is the penalty factor; $U_{i,t}$ is the voltage at node *i*, whose permissible deviation is ±5% of the rated voltage $U_{Ni}$.

To accurately capture the dynamic characteristics of the AC/DC-HDN, this study uses a reward function based on time fluctuations and power information. In DRL, each action not only affects the system state at the current time but also spans a full time period. Specifically, the power at each vertex fluctuates over time, so the reward is calculated by summing the rewards over a typical day for each of the 12 months (12 ∗ 24), reflecting the overall impact of each action. The reward $R_{t}$ is defined as follows:

(14) $$R_{t}\left(a\right)=\sum_{m=1}^{12}\sum_{h=1}^{24}r_{t,m,h}\left(a\right)$$

This paper uses the PPO algorithm, based on the “Actor–Critic” structure, to optimize the expansion planning model. PPO is widely used in reinforcement learning, especially for tasks with discrete action spaces, as it efficiently balances policy optimization with stability. In this study, PPO is applied to optimize the expansion planning of the AC/DC-HDN.

### 3.3. Algorithm Integration

**Figure 6:**
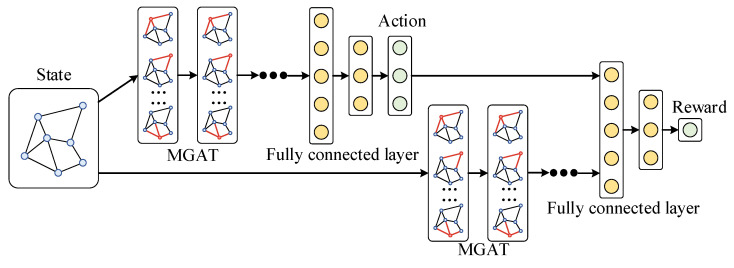
MGAPPO algorithm structure.

To efficiently solve the expansion planning problem for the AC/DC-HDN, the MGAT and PPO algorithms are combined into a unified MGAPPO approach, as shown in Figure 6. The original PPO algorithm uses fully connected neural networks for both the policy and value networks to learn the action policy and state value function. However, the complexity of the network topology and the features of the vertices and edges in the AC/DC-HDN make fully connected networks less effective at capturing the spatial dependencies and global information within the graph structure. To address this, the MGAT is incorporated into the PPO framework, replacing the original networks.

The pseudocode of the MGAPPO algorithm is shown as follows (**Algorithm 1**). The specific algorithm flow of the MGAPPO, when applied to the expansion planning of the AC/DC-HDN, is described below.
**Algorithm 1:** MGAPPO Algorithm1: Initialize the environment.2: Initialize the parameters of the policy network and value network $\theta_{\mathrm{MGAT}},\theta_{\pi },\theta_{Q}$.3: Initialize the optimizer and set the learning rate and other hyperparameters.4: Initialize the experience buffer **B**.**5: For** each training episode ***i***:6:    Obtain the current state $s_{t}$.7:    Use MGAT to extract node and edge embeddings from the graph data.8:    Merge the MGAT-extracted graph embeddings with other features.9:    The policy network $\pi_{\theta }$ generates an action decision $a_{t}$ based on the state $s_{t}$.10:    Execute the action decision $a_{t}$ and obtain the next state $s_{t+1}$.11:    **For** each time step j=1,2…24∗12:12:        Compute the total reward $R_{t}$.13:    Store experience data $\left\{s_{t},a_{t},R_{t},s_{t+1}\right\}$ in the experience buffer **B**.14:    Compute the advantage function.15:    Update the parameters $\theta_{\pi },\theta_{Q}$ of the policy network and value network.16:    Update the parameter $\theta_{\mathrm{MGAT}}$ of the policy network and value network.17: End For18: Output the optimal policy.

First, graph data are constructed based on the AC/DC-HDN’s topology. The vertex features include the DG input, load demand, and main grid power, while the edge features include line type, length, and connection status, forming state *S*. The MGAT then uses an attention mechanism to compute relationships between nodes, generating vertex and edge embeddings that capture spatial dependencies. These embeddings are concatenated to form the embedded state *S’*, which represents the network’s topology and operational characteristics.

In the PPO policy network, the algorithm outputs action A based on state *S’*, which includes decisions on the location of the distributed generation, edge relationships, and type selection. The environment updates the AC/DC-HDN topology and provides an instantaneous reward *R* which guides the agent’s decisions. The PPO value network evaluates the policy and updates both the policy and value networks using the advantage function. A clipped objective function ensures stable learning by limiting policy updates. Through iterations, MGAT refines the graph embedding, while PPO improves the policy. Over time, the networks converge to produce the optimal expansion plan for the distribution network.

The combined MGAT and PPO algorithm offers significant advantages. The MGAT extracts complex node and edge features from the grid topology, capturing spatial relationships, while PPO models the temporal dynamics of grid operation, enabling efficient integration of spatial and temporal information. The joint training mechanism integrates feature extraction and decision optimization, preventing error accumulation and achieving end-to-end global optimization. Reinforcement learning refines the MGAT graph embeddings, aligning them with the planning objectives and improving the overall performance. This hybrid approach leverages the strengths of both the MGAT and PPO, providing an efficient solution for AC/DC-HDN expansion.

## 4. Simulation Results and Analysis

### 4.1. Simulation System Setup

To validate the proposed AC/DC-HDN expansion planning method, three typical distribution networks were selected for simulation:

1. IEEE 33-bus Distribution Network: A small standardized radial network with a simple topology and uniform load distribution, used for initial validation of the optimization algorithm in small networks.
2. IEEE 69-bus Distribution Network: A larger standardized network with more nodes and complex topology, used to test the scalability and applicability of the optimization method in large networks.
3. PG&E 69-bus Distribution Network: A real-world medium-sized network with diverse load characteristics and branching structures, used to evaluate the method’s performance on real data.

These networks, ranging from small and standardized to real-world, cover a wide range of scenarios for performance validation.

To convert the complex distribution network topology into a graph model for optimization, the nodes and topology are simplified as follows:

1. Input Nodes: These include connection points to the higher grid and main transformer nodes, retained as core nodes.
2. Key Load Nodes: These are critical load points, such as industrial or large commercial loads, which are prioritized to ensure that the simplified network captures key load characteristics.
3. Minor Load Nodes: Geographically or electrically similar nodes are merged into an equivalent node, e.g., aggregating residential loads into a regional node. Consecutive minor nodes are simplified into a single equivalent edge.

**Figure 7:**
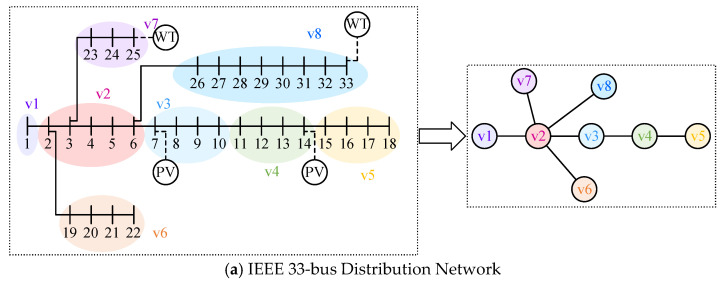

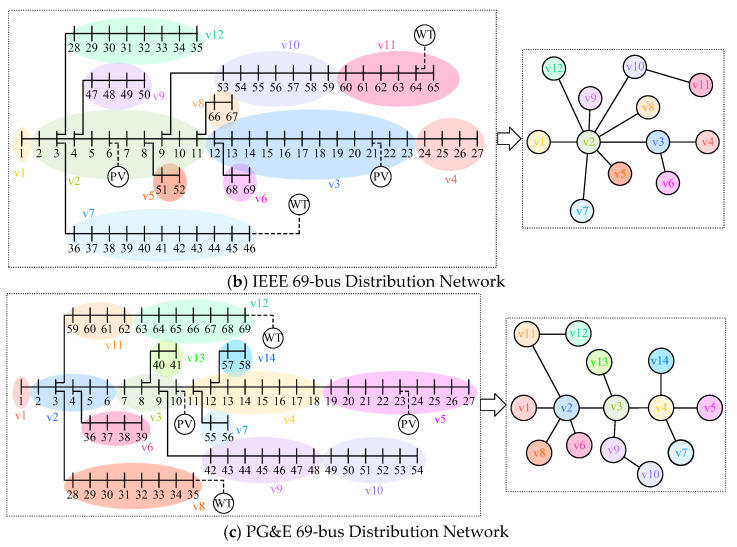
Simplified graph model of typical distribution network.

The graph models for the three distribution networks are shown in Figure 7.

To further validate the proposed method, the AC/DC-HDN is expanded based on the three typical networks. Four nodes in each network are selected for the installation of DGs, including photovoltaic (PV) and wind power units, represented as separate nodes in the graph model.

For the IEEE 33-bus network, two PV plants are installed near nodes 3 and 4, and two wind farms near nodes 7 and 8. In the IEEE 69-bus network, PV plants are placed near nodes 2 and 3, and wind farms near nodes 7 and 11. For the PG&E 69-bus network, PV plants are installed near nodes 3 and 5, and wind farms near nodes 8 and 12.

**Figure 8:**
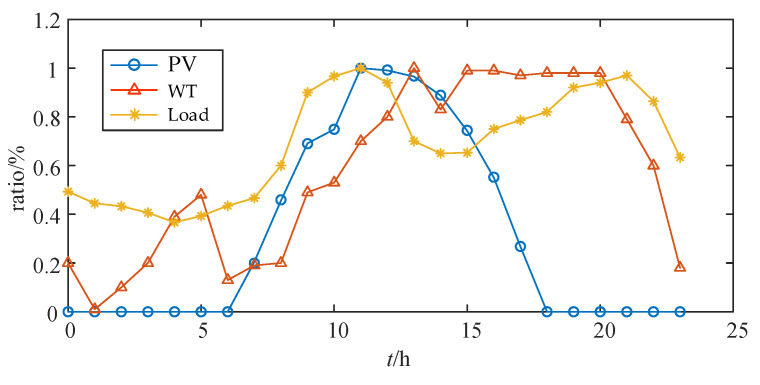
Power output of DGs and load demand variations on a typical day in July.

In this simulation, the data were sourced from historical records of the central mountainous region of Hainan. To accurately model the renewable energy generation and load fluctuations in this area, the maximum likelihood estimation method was used to construct virtual typical daily profiles of the total renewable generation and load for each month across different seasons. The data were then scaled and adjusted to match various distribution network sizes, ensuring the simulation’s applicability to different AC/DC-HDN configurations. Figure 8 presents the variation trends of the power output from photovoltaic and wind power, as well as the load demand, over a 24 h period using data from a typical day in July.

The renewable energy penetration rate is set to 20% (total renewable power/total load), with a PV-to-wind-power-capacity ratio of 2:1. The distributed energy capacities for each network are shown in Table 1.

To simplify the model and better simulate the integration of distributed power sources in practical applications, this study assumes that each distributed power source node is connected to other nodes in the AC/DC-HDN graph by a single edge. Specifically, the following constraint applies to each distributed power source node $v_{\mathrm{DG}}$:

(15) $$\sum_{i\in N}x_{v_{\mathrm{DG}},i}=1$$

Let $x_{v_{\mathrm{DG}},i}$ be a binary decision variable. If the distributed power source node $v_{\mathrm{DG}}$ is connected to node i, then $x_{v_{\mathrm{DG}},i}=1$; otherwise, $x_{v_{\mathrm{DG}},i}=0$. This constraint ensures that each distributed power source node is connected to only one node in the distribution network.

To simplify the optimization and align with standard DC distribution network design principles, the DC distribution lines are assumed to form a tree topology without loops. In other words, the network has no cycles, and the power flows follow unidirectional paths. This constraint is expressed as follows:

(16) $$\sum_{\left(i,j\right)\in \varepsilon }y_{ij}=I-1$$

Let $y_{ij}$ denote the connection between nodes *i* and *j*, with ε as the edge set and *I* as the number of nodes. This constraint ensures an acyclic topology, preventing the formation of loops in the system.

The algorithm parameters must balance optimization efficiency, convergence, and reproducibility. Based on the literature, problem scale, and tuning results, the main parameters for the MGAPPO algorithm are set as shown in Table 2.

### 4.2. Analysis of Algorithm Superiority

**Figure 9:**
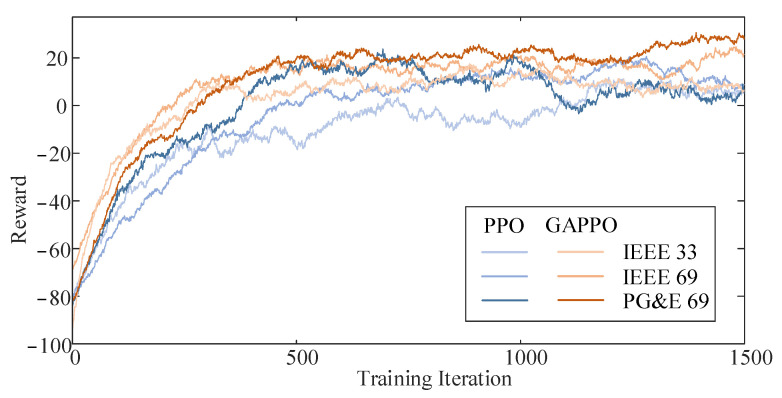
Rewards of different algorithms in three distribution network environments.

In the simulations, the cumulative reward trends of the PPO and MGAPPO algorithms were recorded across different distribution network scenarios to assess their convergence and stability. As shown in Figure 9, in the IEEE 33-node network, MGAPPO’s cumulative reward stabilized after 300 iterations, while PPO only converged after 500 iterations, with significant fluctuations. A similar trend was observed in the IEEE 69-node and PG&E 69-node networks. Additionally, the standard deviation of the reward after 700 iterations, as shown in Table 3, was consistently lower for MGAPPO than for PPO. This indicates that MGAT enhances state representation by extracting graph features, improving the training efficiency and stability.

As shown in Table 4, MGAPPO outperforms PPO in terms of both maximum and average cumulative rewards across all scenarios, highlighting that MGAT better captures the spatiotemporal correlations of the distribution network’s topology and operations.

### 4.3. Analysis of Simulation Results

**Figure 10:**
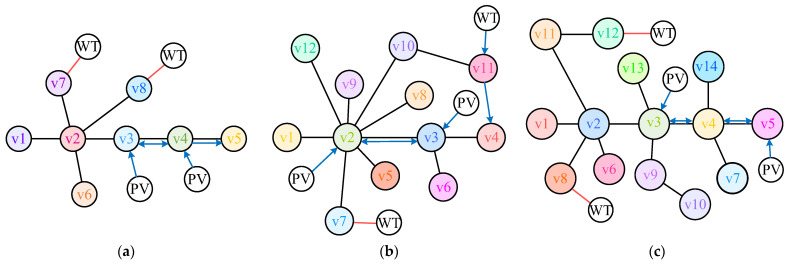
Optimization results. (**a**) IEEE 33-node distribution network optimization results, (**b**) IEEE 69-node distribution network optimization results, (**c**) PG&E 69-node distribution network optimization results.

Figure 10 shows the optimized schemes for three distribution networks, including the placement of distributed energy sources and the optimized line topology. In this figure, the blue line represents the DC line and the red line represents the AC line.

In the IEEE 33-node network, the MGAPPO algorithm places two PVs near nodes 3 and 4 and two WTs near nodes 7 and 8, where nodes 3 and 4 are connected through a bidirectional DC line. It also optimizes the AC/DC-HDN line to reduce power loss.

For the IEEE 69-node network, MGAPPO uses spatial information to efficiently reduce redundant lines and optimize the AC/DC-HDN lines. PVs are connected to node 2 and node 3 through unidirectional DC lines, and nodes 2 and 3 are connected by bidirectional DC lines. One WT is connected to node 7 via an AC line, and another is connected to node 11 via a unidirectional DC line, extending to node 4.

In the PG&E 69-node network, the MGAPPO plan better aligns with engineering needs by placing distributed energy sources in load-dense areas and minimizing unnecessary AC/DC line overlap. WTs are connected to nodes 12 and 8, PVs are connected to nodes 3 and 5, and nodes 3 and 5 are connected through bidirectional DC lines.

**Figure 11:**
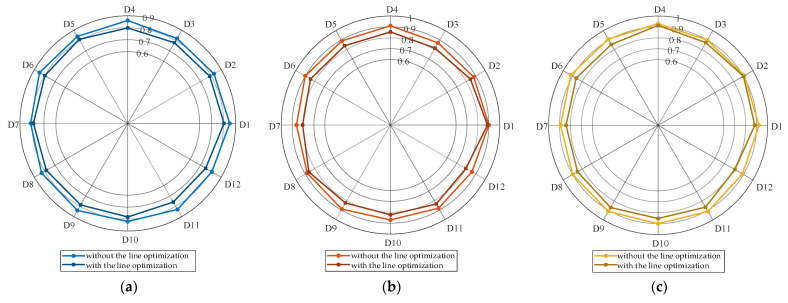
Comparison of power supply of main grid with and without line optimization. (**a**) IEEE 33-node distribution network, (**b**) IEEE 69-node distribution network, (**c**) PG&E 69-node distribution network.

Figure 11 compares the renewable energy absorption capacity of the AC/DC-HDN with and without the application of line optimization results, with DGs already being integrated into the system. In the radar chart, D1 to D12 represent 12 typical days, while the scale values (0, 0.2, 0.4, …) indicate the normalized power supplied by the upper-level grid. A lower value signifies a higher renewable energy absorption capacity.

As shown in the figure, after expansion, the power supplied by the main grid decreases across all typical days for the three distribution networks. The optimized curves contract toward the center, indicating improved local absorption of photovoltaic and wind power and reduced dependence on the main grid. Notably, on certain days, the supply power drops significantly after optimization, demonstrating that the expansion planning strategy effectively enhances local consumption. Overall, the renewable energy consumption capacity increases by 3.64%, 3.31%, and 2.77% for the IEEE 33-bus, IEEE 69-bus, and PG&E 69-bus networks, respectively, reflecting the increase in the actual energy that is consumed from renewable sources. This optimized topology improves the ability to accommodate photovoltaic and wind power generation by reducing the reliance on the main grid, thus facilitating a greater renewable energy consumption capacity.

**Figure 12:**
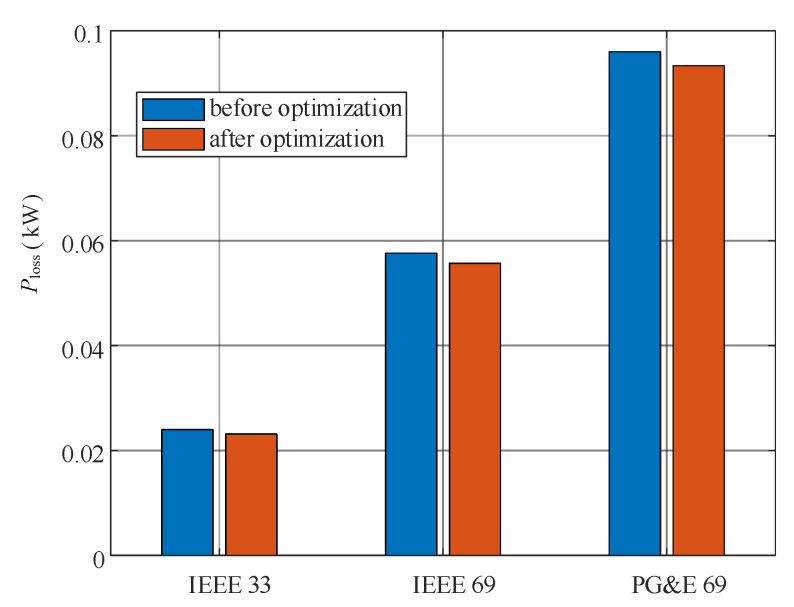
Comparison of annual network loss before and after optimization.

Regarding power losses, the expansion method also leads to improvements. Figure 12 presents a comparison of annual network active power losses before and after optimization. By strategically planning the layout of lines, the power flow is optimized, shortening transmission paths and balancing the current distribution, thereby reducing power losses. Specifically, line losses decrease by 2.42% in the IEEE 33-bus network, 2.21% in the IEEE 69-bus network, and 1.84% in the PG&E 69-bus network. These reductions not only improve system efficiency but also enhance economic and operational reliability.

The visualization of the optimization plan clearly demonstrates the improvements in distribution network planning using the MGAPPO algorithm. In all scenarios, MGAPPO efficiently plans the lines and connection points while considering load distribution and system topology constraints. The resulting plan is both practical and optimized for renewable energy consumption considerations.

## 5. Conclusions

This paper presents an expansion planning method for AC/DC-HDNs that incorporates spatial–temporal correlations, addressing the challenges posed by increasing renewable energy penetration and rising power demand. By integrating a graph-theory-based network model with load dynamics and distributed energy source distribution, the proposed optimization method enhances the efficiency and flexibility of planning while improving the renewable energy consumption capacity.

The simulation results show that the proposed method outperforms traditional optimization techniques in various scenarios, efficiently planning lines and connection points, reducing power losses, and ensuring system reliability. This multi-objective decision framework offers significant practical value, providing a novel approach for expanding AC/DC-HDNs. Future work may focus on applying this method to larger, more complex networks and integrating it with other optimization algorithms. Additionally, we plan to further refine and validate our approach in an actual distribution network to enhance its practical applicability.

To clarify, while the term “Expansion Planning” is often associated with long-term energy planning and infrastructure development, this study focuses on short-term operational optimization aimed at improving renewable energy absorption within a one-year planning horizon. The analysis is primarily concerned with network performance improvements in the near term, rather than the long-term infrastructure growth that is typically addressed in expansion planning. However, the algorithm proposed in this study can be applied to long-term expansion planning scenarios as well.

## Figures and Tables

**Table 1:** DG access capacity for each distribution network (PV: Wind = 2:1).

Network	Total System Load (MW)	Renewable Energy Penetration Rate	Total New Energy Power (MW)	Number of PV Generation Units	Capacity per PV Unit	Number of WT Generation Units	Capacity per WT Unit
IEEE 33-bus network	3.7	20%	0.74	2	0.245	2	0.125
IEEE 69-bus network	4.8	20%	0.96	2	0.32	2	0.16
PG&E 69-bus network	6.5	20%	1.3	2	0.435	2	0.215

**Table 3:** Standard deviation of reward values after 700 training iterations.

Network	PPO	MGAPPO
IEEE 33-bus network	2.8501	1.5764
IEEE 69-bus network	2.5219	1.9293
PG&E 69-bus network	4.7833	2.1056

**Table 2:** Main parameter settings for MGAPPO.

Parameter	Value	Remarks
PPO Initial Learning Rate	0.0003	Adaptive learning rate decay
PPO Clipping Parameter	0.2	Limits the magnitude of policy updates
MGAT Layers	3	Each layer contains multiple attention heads
Attention Heads	8	Extracts multi-level relational information
Embedding Dimension	64	Node and edge feature embedding dimension
Dropout Probability	0.2	Prevents overfitting
Activation Function	Leaky ReLU	Captures non-linear characteristics
Adam Optimizer Learning Rate	0.0003	Consistent optimization strategy

**Table 4:** Maximum and average reward values after 700 training iterations.

Network	Maximum Values		Average Values	
	PPO	MGAPPO	PPO	MGAPPO
IEEE 33-bus network	13.7466	18.1207	5.7294	9.1213
IEEE 69-bus network	20.8592	23.3976	10.2698	18.9743
PG&E 69-bus network	23.2434	25.9898	11.6874	20.2618

## Data Availability

Data are contained within the article.
